# Supplementary material for: Physics-informed data-driven discovery of constitutive models with application to strain-rate-sensitive soft materials
Source: Comput Mech. 2024 Jun 17;77(2):357–86. doi: 10.1007/s00466-024-02497-x (PMC12963190; doi:10.1007/s00466-024-02497-x)
Supplement: Supplementary file 1 — (pdf 663 KB) [file 466_2024_2497_MOESM1_ESM.pdf]

PHYSICS-INFORMED DATA-DRIVEN DISCOVERY OF CONSTITUTIVE MODELS WITH APPLICATION TO STRAIN-RATE-SENSITIVE SOFT MATERIALS

SUPPLEMENTARY MATERIAL

Kshitiz Upadhyay*
Department of Mechanical and Industrial Engineering
Louisiana State University
Baton Rouge, LA 70803, USA
kshitizu@lsu.edu | ORCID ID: 0000-0002-0800-4888

Jan N. Fuhg
Sibley School of Mechanical and Aerospace Engineering
Cornell University
Ithaca, NY 14850, USA
jf853@cornell.edu | ORCID ID: 0000-0002-5986-3770

Nikolaos Bouklas
Sibley School of Mechanical and Aerospace Engineering
Center for Applied Mathematics
Cornell University
Ithaca, NY 14850, USA
nb589@cornell.edu | ORCID ID: 0000-0002-3349-5914

K.T. Ramesh
Department of Mechanical Engineering, and Hopkins Extreme Materials Institute
Johns Hopkins University
Baltimore, MD 21210, USA
ramesh@jhu.edu | ORCID: 0000-0003-2659-4698

SM1 Data-driven Model Predictions in the Confined Tension Regime

In the Section 5.1 of the main manuscript on "The $\widetilde{\mathcal{M}}_{\text{vol}}$ Surrogate Model Under Hydrostatic (Confined Uniaxial) Loading", two data-driven constitutive models, the present physics-informed surrogate model and a classical mapping model, were trained using stress–strain data under the confined compression deformation mode. A small dataset comprising of 26 data-points was considered in the training process. Figure SM1 shown below plots the stress predictions of these data-driven models in the uniaxial testing regime (i.e., compression + tension) when they are trained using a much larger training dataset of 201 data-points.

*Corresponding author

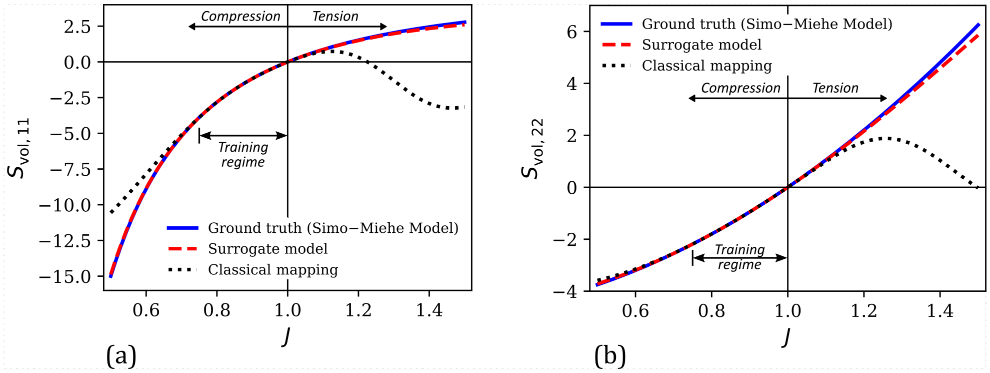

Figure SM1: Stress predictions of data-driven models in the uniaxial testing regime (i.e., compression + tension) when they are trained using a training dataset of 201 data-points.
